# Thermal Degradation Kinetics Analysis of Polymer Composite Electrolyte Membranes of PEVOH and PBT Nano Fiber

**DOI:** 10.3390/polym14030537

**Published:** 2022-01-28

**Authors:** Sheng-Jen Lin, Gwomei Wu

**Affiliations:** Institute of Electro-Optical Engineering, Chang Gung University, Chang Gung Memorial Hospital, Taoyuan 333, Taiwan; aa691010@yahoo.com.tw

**Keywords:** thermal degradation kinetics, polymer composite, electrolyte membrane, EVOH, PBT, nano fiber

## Abstract

The thermal degradation kinetics of high-performance polymer composite electrolyte membranes were investigated by thermal gravimetric analysis in this study. The novel porous polymer composite membranes were fabricated by crosslinking poly (ethylene-co-vinyl alcohol) (EVOH) with polybutylene terephthalate (PBT) nano fiber. The PBT nano-scale fiber non-woven cloth was first prepared by the electrospinning method to form a labyrinth-like structure, and the crosslinking was carried out by filtering it through a solution of EVOH and crosslinking agent triallylamine using the Porcelain Buchner funnel vacuum filtration method. The PBT–EVOH composite membranes with various crosslinking agent ratios and ethylene carbonate/dimethyl carbonate (EC/DMC) immersion times were investigated for their thermal stability and ionic conductivity. The results showed that the higher crosslinking agent content would lower the crystallinity and enhance thermal stability. The thermal degradation activation energy was dramatically increased from 125 kJ/mol to 340 kJ/mol for the 1.5% crosslinking agent content sample at 80% conversion. The triallylamine crosslinking agent was indeed effective in improving thermal degradation resistivity. The best ionic conductivity of the polymer composite membranes was exhibited at 5.04 × 10^−3^ S cm^−1^ using the optimal weight ratio of EVOH/PBT composite controlled at 1/2. On the other hand, the EC/DMC immersion time was more effective in controlling the R_b_ value, thus the ionic conductivity of the membranes. A higher immersion time, such as 48 h, not only gave higher conductivity data but also provided more stable results. The triallylamine crosslinking agent improved the membrane ionic conductivity by about 22%.

## 1. Introduction

A lithium-ion rechargeable battery is a remarkable energy-storage system, attributed to the excellent energy density and high working voltage [1]. However, the electrochemical device’s safety has always been of great concern as one of the most important issues that could limit its wider use in mobile electronic products and electric vehicles. Damage has been reported from time to time through battery explosions of products even those produced by many renowned international companies. It has been noted that high-performance polymer composite electrolyte membranes may play important roles in battery structures, not only to provide high ionic conductivity but also to construct a thin and tough interface between anodes and cathodes [2,3,4,5]. Their structures and electrochemical characteristics can be crucial for energy storage and energy conversion [6]. Nevertheless, when compared to the traditional non-woven cloth membrane separators, the use of organic/inorganic electrolyte membranes can be limited by some key factors, such as a complicated synthesis route [7], low ionic conductivity [8], low mechanical strength [9], difficulty in mass production [10], environmental solvent pollution [11], and high cost [12]. These issues drastically limit the applications in battery systems.

An ideal membrane separator should be designed to safely isolate the anode and cathode, and also to supply adequate ionic transport during the charge and discharge processes [13]. Conventional polyethylene, polypropylene and their composites have usually been selected as the base materials for commercial separators due to the cost and processability. However, a battery can be used under high power charge or discharge processes, and the rapid or abnormal current loading may induce transient temperature climbing. Transient high temperature could easily induce thermal shrinkage for those polyolefin-based separators and result in a battery short circuit. In order to resolve these undesirable problems such as electrolyte leakage, related low ionic conductivity and thermal shrinkage, it has been desirable to develop polymer composite electrolyte membranes with improved characteristics to replace the conventional separators. However, the poor ionic conductivity and low mechanical integrity have still restricted the development in rechargeable battery systems [14,15]. Several polymeric materials have been studied for their thermal shrinkage and electrochemical performances, including Al_2_O_3_/poly(vinylidene fluoride-hexafluoropropylene) composite separators for lithium-ion batteries [16], the polyacrylonitrile system [17], and a gelled polymer electrolyte with inorganic fillers [18,19]. For example, the pristine polyethylene has a significant shrinkage percentage of 86% at 140 °C. Liao et al. developed Al_2_O_3-_ and SiO_2_-based solid polymeric electrolyte membrane separators and achieved lower dimensional shrinkage of 65% and 63%, respectively [20]. Although some polymer membranes showed good ionic conductivity in the range of 10^−3^ S cm^−1^, poor mechanical strength and thermal stability were still observed. This would result in breaking and cracking of electrolyte membranes during the battery fabrication and the charge/discharge procedures. The internal short-circuits might cause an irreversible reaction and inhibit the battery performance.

Polybutylene terephthalate (PBT) is a semi-crystalline engineering thermoplastic material that shows high mechanical strength, low molding shrinkage and good thermal resistance [21,22]. It can be processed by several available techniques and is readily dissolvable in methylene chloride. This creates a possible route for electrospinning into microporous non-woven structures [23,24]. The porous polymer composites are very important and have been widely used in many practical applications, such as fibrous porous media [25]. The effective electrolyte diffusion through charged porous media has been also validated by a fractal model. The experimental data yielded satisfying agreement with semi-analytical model results [26]. The PBT non-woven cloth could thus result in having properties with very high permeability and binding capacity for various applications. The electrospinning method has been developed for several years to produce nanofibers for industry [27,28,29]. The rheological properties of the polymer solutions showed critical effects on the essential electro-spinnability. It was also demonstrated to have produced a dye-sensitized solar cell to harvest solar energy. Xiao, et al. developed a layer-by-layer structure for electrospun poly (vinyl alcohol) nanofibers with ZrO_2_ nanoparticles. Their polymer composites exhibited improved mechanical tensile strength up to 14.5 MPa [30]. On the other hand, ethylene vinyl alcohol (EVOH) copolymer is hydrophilic with high inter- and intra-molecular cohesive energy. The incorporation can be utilized to improve electrolyte uptake by the plasticization effect of the copolymer [31]. This copolymer has been recently reported to be electrospun to obtain hybrid nanocomposites. The resultant fiber mats were further annealed to promote inter-fiber coalescence [32].

In order to obtain good ionic conductivity with higher thermal resistance for polymer composites, we present the synthesis and characteristic properties of PBT–EVOH polymer composite electrolyte membranes in this study. A higher thermal stability should improve the life-cycle for an energy storage and/or energy conversion system [33]. The PBT nanofiber non-woven cloth was firstly prepared by electrospinning, then crosslinked [34] with EVOH using various crosslinking agent ratios and membrane immersion times. The thermal stability was analyzed by the Ozawa method [35,36]. The derived activation energy can reveal the difficulty in thermal degradation reaction of the materials. Meng, et al. studied the Flynn–Wall–Ozawa methods to calculate the activation energy of 1-butyl-2,3-dimethylimidazolium nitrate [37]. Canakci also investigated the activation energies of synthesized compounds from the slope of the plot vs. 1/T. Therefore, the experimental data could be followed for each stage of the thermal degradation that occurred at different rates. The kinetics relationship between the fractional conversion and the activation energy was then analyzed [38]. The resulting polymer composite membranes would show good ionic conductivity with improved thermal stability. The ionic conductivity was measured by AC impedance spectroscopy. The structural properties and electrochemical characteristics have been systematically studied and are discussed in this paper. The ionic conductivity could be improved to 5.04 × 10^−3^ S cm^−1^ and the thermal degradation activation energy was increased to 340 kJ/mol. The crosslinking agent should have played a role in lowering the crystallinity and enhancing the thermal stability.

## 2. Materials and Methods

**Figure 1:**
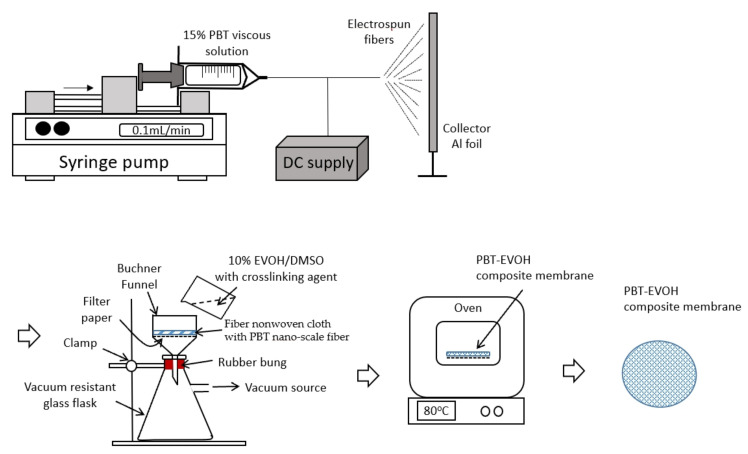
Schematic illustration of the preparation of polybutylene terephthalate–ethylene vinyl alcohol (PBT–EVOH) composite membranes.

The polymer composite electrolyte membranes were fabricated by crosslinking poly (ethylene-co-vinyl alcohol) (Chang Chun Petrochemical, Taipei, Taiwan) and polybutylene terephthalate (Shinkong Synthetic Fibers, Taipei, Taiwan). The PBT chips were firstly dried and dissolved in methylene chloride (Sigma-Aldrich, St. Louis, MO, USA) to form a 15 wt.% viscous solution. The fiber non-woven cloth with PBT nano-scale fiber was then prepared by an electrospinning method to form a labyrinth-like structure. Further crosslinking was carried out by filtering through a 10 wt.% EVOH dissolved in dimethyl sulfoxide (DMSO, Sigma-Aldrich) solution using a different crosslinking agent (triallylamine, Sigma-Aldrich) contents at 0, 0.2 and 1.5%, with 0.1% azobisisobutyronitrile (AIBN, Sigma-Aldrich). Finally, the composite membrane cloth samples were finished with good binding membrane characteristics by using the Porcelain Buchner funnel vacuum filtration method. The weight ratio of the EVOH versus PBT fiber after filtration was nominally controlled at 1:2. The prepared polymer composite membranes were shaped into round sheets and then immersed in ethylene carbonate and dimethyl carbonate (EC/DMC = 1:1 (*v*/*v*) 1 M) at 25 °C for a specified period of time. The samples were then assembled between a pair of SS316 stainless steel electrodes in an argon-filled glove box (Braun, Germany; working condition, H_2_O: 3 ppm, O_2_: 1 ppm). Figure 1 shows the schematic illustration of the preparation steps of the PBT–EVOH composite membranes.

The surface morphology study of the prepared PBT–EVOH polymer composite membrane samples was performed using a JEOL JSM-5600LV scanning electron microscope (SEM). The X-ray diffraction (XRD) measurements were carried out using a Rigaku MultiFlex diffractometer, equipped with a monochromator and a Cu target tube. The radiation wavelength λ was 0.154 nm. The continuous scans were conducted from 10° to 80°, with a step size of 0.05° and a scan rate of 10°/min. Differential scanning calorimetry (DSC) thermal analysis was performed by using a Perkin Elmer Pyris 7 DSC system. The measurements were taken by heating ~5 mg samples from 25 °C to 300 °C, in N_2_ atmosphere. Thermogravimetric analysis (TGA) experiments were carried out using a Perkin Elmer Pyris 7 TGA system. The measurements were made from 25 °C to 500 °C, also under N_2_ atmosphere. In this study, for the the thermal degradability of PBT–EVOH composite films we followed the Ozawa method to determine the corresponding thermal degradation activation energy of the polymer. The Ozawa equation is listed here as [39,40]:

−*logA_1_* − *0.4567* (*Ea/RT_1_*) = −*logA_2_* − *0.4567* (*Ea/RT_2_*) (1)

where *Ai* is the heating rate, *Ea* is the activation energy, and *T* is the temperature in Kelvins.

The AC impedance method was employed to measure the ionic conductivity. The PBT–EVOH polymer composite electrolyte membranes had a contact surface area of 1.29 cm^2^. The AC impedance measurements were carried out by AutoLab from Eco Chemi, with a computer program. In addition, the frequency range from 100 Hz to 100 kHz was recorded. The following equation has been used to calculate the ionic conductivity σ in this study.

(2) $$\sigma =\frac{L}{R_{b}\times A}$$

where, *L* and *A* are the thickness and area of the polymer composite membranes, respectively. *R_b_* was derived from the Nyquist plot of the AC impedance analysis [41].

## 3. Results and Discussion

### 3.1. Surface Morphology

**Figure 2:**
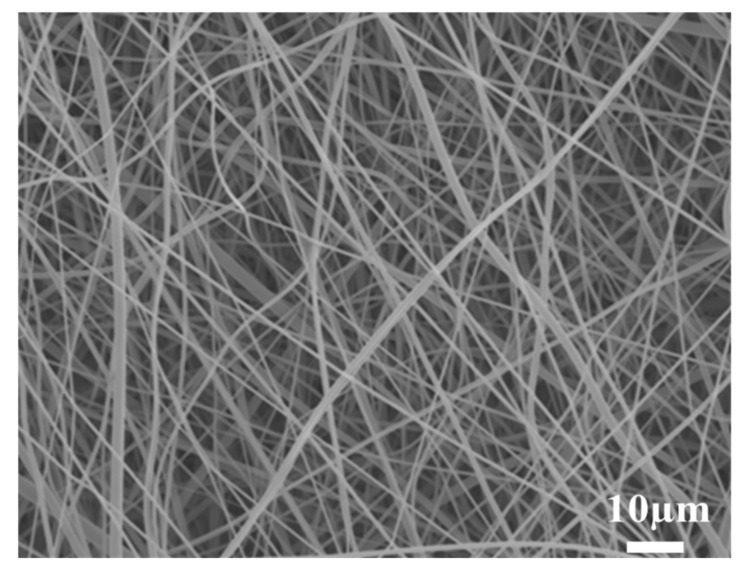
Scanning electron microscopy (SEM) micrograph of the PBT non-woven formed in a labyrinth-like structure.

**Figure 3:**
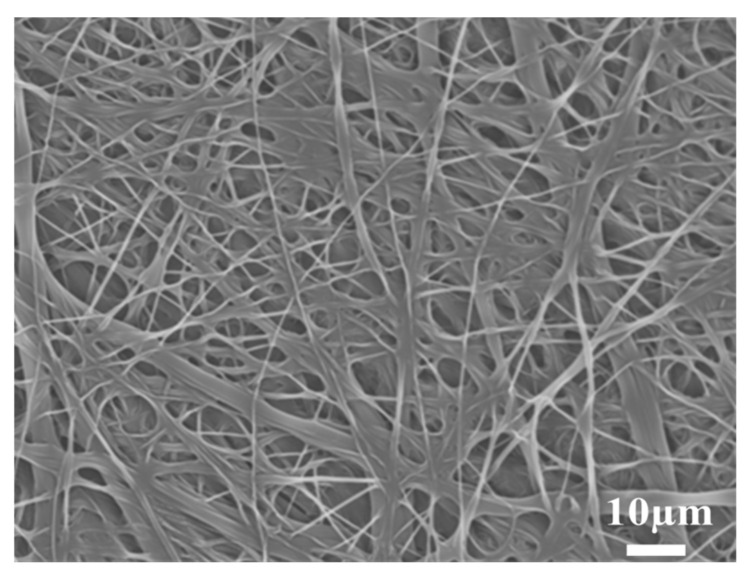
SEM micrograph of the PBT–EVOH polymer composite film, prepared with the triallylamine crosslinking agent ratio of 1.5%.

The SEM micrograph of the PBT non-woven cloth that was prepared by the electrospinning method is shown in Figure 2. It can be observed that the non-woven cloth with numerous PBT fibers was formed in a labyrinth-like structure. It has a lot of pores for potential electrolyte transport. The fiber size was mainly in the range of 50~500 nm. Figure 3 shows the SEM micrograph of the PBT–EVOH composite films with the triallylamine crosslinking agent ratio of 1.5%. It shows that the PBT nano-scale fibers were all well covered by EVOH and the micro-pores were also distributed uniformly. This is indeed beneficial for electrochemical applications due to more electronic transport channels. The gas aggregation near the polymer composite membrane surface could be also reduced during the battery charge/discharge process.

### 3.2. Thermal Degradation Stability

**Figure 4:**
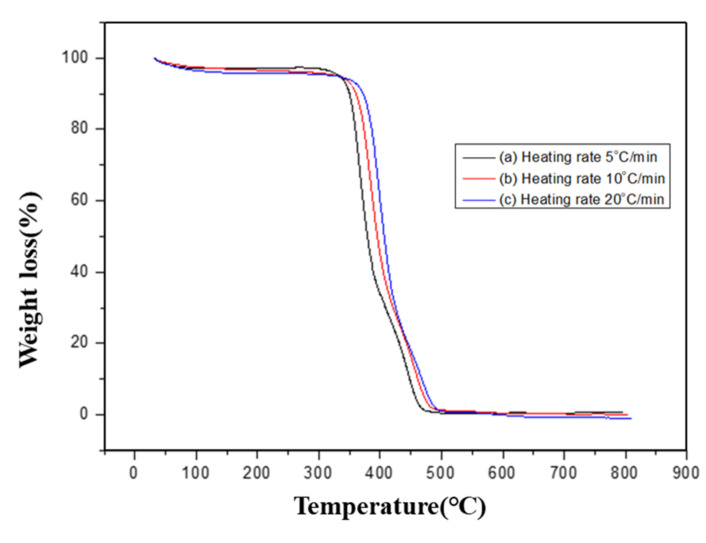
Thermogravimetric analysis (TGA) scans of the PBT–EVOH polymer composite film using 0.2% triallylamine crosslinking agent.

The thermal degradability of PBT–EVOH polymer composite films was investigated using Ozawa method to determine the thermal degradation activation energy. Figure 4 shows an example of the TGA thermogravimetric scans of the PBT–EVOH composite film using 0.2% triallylamine crosslinking agent. Three different heating rates were employed.

**Figure 5:**
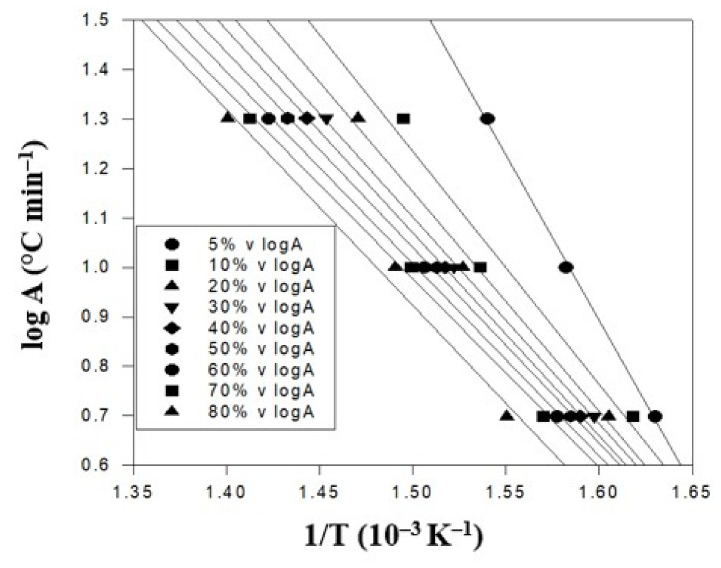
TGA thermal degradation analysis results of the PBT–EVOH polymer composite film using no triallylamine crosslinking agent.

In the study, including 5, 10, and 20 °C/min. In general, under the same weight loss percentage or degradation conversion percentage, a higher heating rate resulted in a higher conversion temperature. This trend was in good agreement with the thermal analysis models in the literature [39]. At the weight loss of 50%, the higher heating rate of 20 °C/min was achieved at 425 °C. It was lowered to 388 °C when the middle heating rate of 10 °C/min was used. It was further reduced to 358 °C for the lowest heating rate of 5 °C/min. This was caused by the complicated thermal degradation kinetics of the polymer composite materials and should be further analyzed. Figure 5 displays the TGA thermal degradation analysis results of the PBT–EVOH composite film, prepared using 0% or no triallylamine crosslinking agent. The derived corresponding thermal analysis results are listed in Table 1. The thermal degradation activation energy *Ea* ranged from 211 kJ/mol at 5% conversion to 125 kJ/mol at 80% conversion. The correlation coefficient *R^2^* was high at 0.965~0.999, nearly 1.

**Figure 6:**
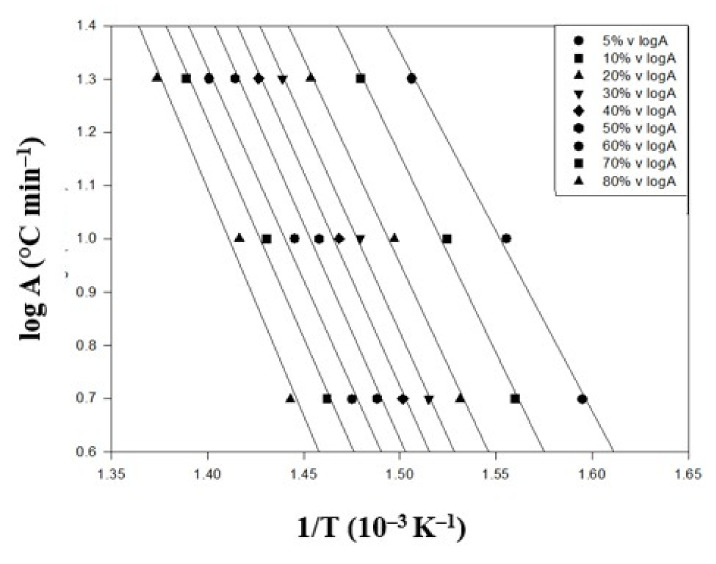
TGA thermal degradation analysis results of the PBT–EVOH polymer composite film using 0.2% triallylamine crosslinking agent.

The TGA thermal degradation analysis results of the PBT–EVOH polymer composite film, prepared using 0.2% triallylamine crosslinking agent, are shown in Figure 6. The same three different heating rates were employed in this analysis. The conversion temperature followed the same increasing trend with the heating rate results in Figure 5. Thus, the higher the heating rate, the higher the degradation temperature data that were observed. However, all the conversion temperature data increased further, indicating more resistance to the thermal degradation. The derived corresponding thermal analysis results of the 0.2%-triallylamine-crosslinking PBT–EVOH polymer composite film are shown in Table 2. It can be seen that the thermal degradation activation energy Ea ranged from 209 kJ/mol at 5% conversion to 237 kJ/mol at 80% conversion. The activation energy increased from the PBT–EVOH polymer composite film sample without using triallylamine crosslinking agent. Thus, the application of triallylamine crosslinking agent was quite effective in improving the thermal degradation resistivity of the polymer composites. The correlation coefficient R^2^ has been also high at 0.982~0.999, nearly 1.

**Figure 7:**
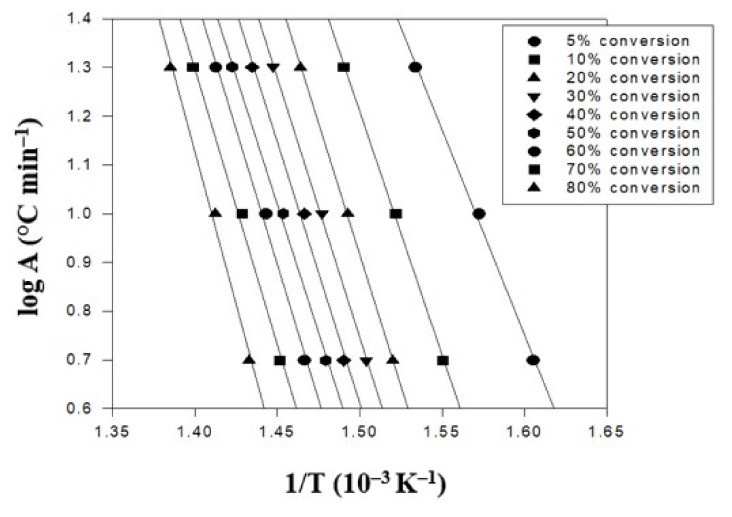
TGA thermal degradation analysis results of the PBT–EVOH polymer composite film using 1.5% triallylamine crosslinking agent.

Finally, the triallylamine crosslinking agent content was increased to 1.5% for further evaluation. Thus, the TGA thermal degradation analysis results of the PBT–EVOH polymer composite film, prepared using 1.5% triallylamine crosslinking agent, are presented in Figure 7. Again, the same three heating rates were employed in this analysis. The conversion temperature followed the same increasing trend with the heating rate results in Figure 5. Although all the conversion temperature data increased more than the no-triallylamine-crosslinking PBT–EVOH polymer composite film sample, they are not much higher than the 0.2%-triallylamine-crosslinking PBT–EVOH polymer composite film sample. However, the slope data were significantly higher. The derived corresponding thermal degradation analysis results of the 1.5%-triallylamine-crosslinking PBT–EVOH polymer composite film are shown in Table 3. The thermal degradation activation energy *Ea* ranged from 259 kJ/mol at 5% conversion to 340 kJ/mol at 80% conversion. It is thus evidenced that the activation energy increased well from the PBT–EVOH polymer composite film sample without using triallylamine crosslinking agent. The application of triallylamine crosslinking agent was indeed very effective in improving the thermal degradation resistivity of the polymer composite film. The correlation coefficient *R^2^* was also high at 0.993~0.999, nearly 1.

In brief summary, the TGA thermal degradation analysis results showed that the higher the crosslinking agent content, the higher the activation energy that could be obtained. It was dramatically increased from 125 kJ/mol with no crosslinking content to 340 kJ/mol while using 1.5% crosslinking agent content at the 80% conversion. It has been also proved that triallylamine is an effective crosslinking agent to enhance the PBT–EVOH polymer composite film’s thermal stability.

### 3.3. Differential Scanning Calorimetry (DSC) Analysis

**Figure 8:**
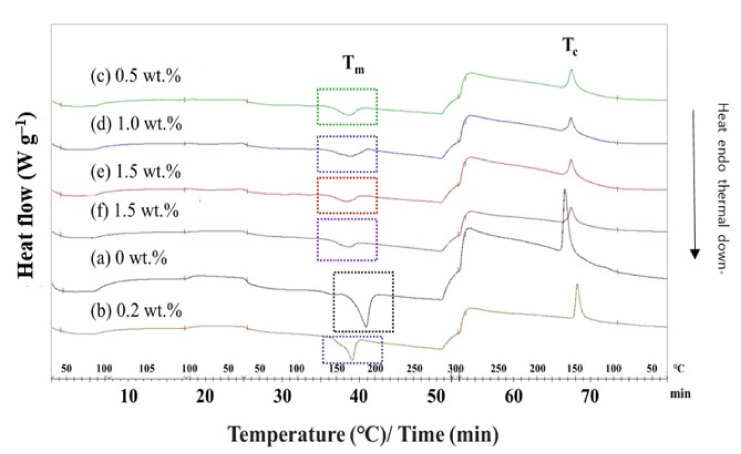
DSC thermal calorimetry scans of the PBT–EVOH polymer composite film.

The DSC analysis of the PBT–EVOH polymer composite films was undertaken in terms of the heating and cooling rate of 10 °C/min to determine the melting temperature T_m_ and the crystallization temperature T_c_. The DSC thermal calorimetry scan results of the PBT- EVOH polymer composite films are displayed in Figure 8. The results showed that both the T_m_ and enthalpy (∆H) were significantly decreased with the addition of higher triallylamine crosslinking agent content. The T_m_ was reduced from 187 °C for the PBT–EVOH polymer composite film sample without using triallylamine crosslinking agent to 170 °C for the 1.5%-triallylamine-crosslinking PBT–EVOH polymer composite film. The data are slightly lower than the values reported for pure PBT due to the incorporation of EVOH [21]. The endothermic enthalpy was also decreased from –73.7 J/g for the polymer composite film using no triallylamine crosslinking agent to –24.6 J/g for the 1.5%-triallylamine-crosslinking polymer composite film. In addition, the T_c_ was decreased from the addition of the triallylamine crosslinking agent. It was reduced from 165 °C for the PBT–EVOH polymer composite film sample without using the triallylamine crosslinking agent to 156 °C for the 1.5%-triallylamine-crosslinking PBT–EVOH polymer composite film. It has been suggested that the crosslinking agent might have decreased the crystallinity of the PBT–EVOH polymer composite film.

### 3.4. X-Ray Diffraction (XRD) Analysis

**Figure 9:**
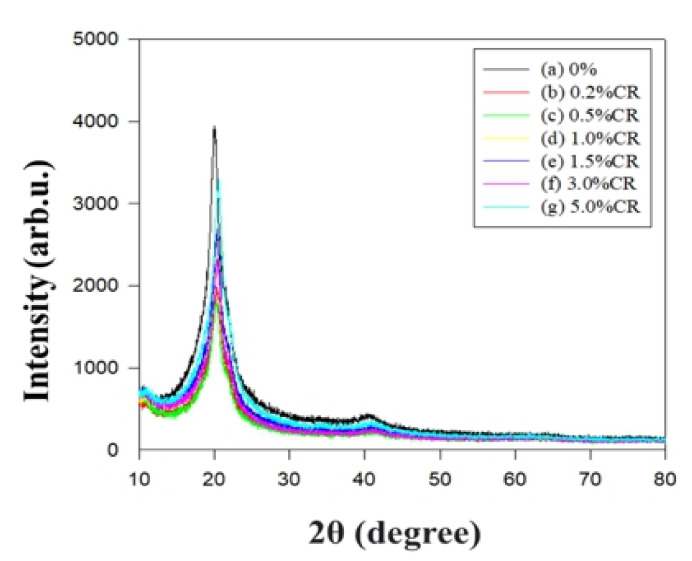
X-ray diffraction (XRD) scans of the PBT–EVOH polymer composite films.

XRD analysis of the PBT–EVOH polymer composite films was conducted using a scan rate of 10°/min at room temperature. Figure 9 shows the XRD diffraction scans of the different PBT–EVOH polymer composite films with various triallylamine crosslinking agent ratios. Typical peaks could be observed at the 2θ of 20° for all the scans. The peak intensity slightly decreased with the increasing crosslinking agent content. This suggested that the crosslinking agent would have decreased the crystallinity of the PBT–EVOH polymer composite film. This is likely beneficial for an ionic transport application due to the lower barrier effect from the non-crystalline part of the polymer composite film. The lower crystallinity film could also exhibit the higher ionic conductivity. In addition, both the PBT and EVOH materials are relatively safe because they have no toxicity to be widely used in many fields, including food packaging applications [21,30].

### 3.5. Ionic Conductivity Analysis

Table 4 summarizes the ionic conductivity analysis results of the 15%-PBT and 10%-EVOH polymer composite electrolyte membranes at 25 °C. The polymer composite membranes had been immersed in ethylene carbonate and dimethyl carbonate for different periods of time. The immersion time has an impact on the polymer composite electrolyte membrane performances. Typically, the R_b_ values for the PBT–EVOH crosslinked polymer composite electrolytes are in the order of 1~10 Ω and they are more dependent upon the electrolyte solution immersion time for the films. The shorter immersion time of 24 h was not enough to provide satisfactory electrolyte absorption for the polymer composite films. The electrical resistance obtained was higher and the samples exhibited lower conductivity. The trend for crosslinking agent ratio on conductivity was also not clearly revealed. On the other hand, at a higher immersion time, such as 48 h, it not only gave higher conductivity data but also provided more stable/predictable results. The ionic conductivity values are in the order of 10^−3^~10^−4^ S cm^−1^. The results were comparable with the conductivity data reported in the literature, such as 5.1 × 10^−4^ S cm^−1^ measured at 60 °C by Zhan et al. [3] and 5.2 × 10^−3^ S cm^−1^ at room temperature by Aziz et al. [6]. In this study, it was experimentally observed that the ionic conductivity of the PBT–EVOH polymer composite electrolyte membrane with the 1.5% crosslinking agent ratio and 48 h immersion time could exhibit the best *σ* of 5.04 × 10^−3^ S cm^−1^. Thus, the addition of triallylamine crosslinking agent improved the sample’s ionic conductivity from 4.12 × 10^−3^ S cm^−1^ by about 22%.

## 4. Conclusions

The PBT–EVOH polymer composite electrolyte membranes were prepared well with different crosslinking agent ratios and EC/DMC immersion times for this study. This was achieved by the combination of the electrospinning method and using a Porcelain Buchner funnel vacuum filtration method. The results showed that the higher crosslinking agent content would lower the crystallinity and enhance the thermal stability. The TGA thermal degradation activation energy was dramatically increased from 125 kJ/mol for the no crosslinking content sample to 340 kJ/mol for the 1.5% crosslinking agent content sample at the 80% conversion. The application of triallylamine crosslinking agent was indeed very effective in improving the thermal degradation resistivity of the polymer composite film. The correlation coefficients *R^2^* were all near 1. The ionic conductivity of the polymer composite electrolyte membrane could exhibit *σ* of 5.04 × 10^−3^ S cm^−1^ for the 1.5% crosslinking agent content sample. On the other hand, the EC/DMC immersion time was more effective in controlling the *R_b_* values, and thus the ionic conductivity results of the polymer composite membranes. A higher immersion time, such as 48 h, not only gave higher conductivity data but also provided more stable/predictable results. Thus, a suitable immersion time is highly recommended for the PBT–EVOH polymer composite membranes. The addition of triallylamine crosslinking agent experimentally improved the polymer composite’s ionic conductivity from 4.12 × 10^−3^ S cm^−1^ by about 22%. This investigation should help to pave the way for the successful development of polymer composite electrolyte membranes with improved thermal stability and good ionic conductivity for rechargeable battery systems. In addition, the ionic transport in relation to the microporous polymer composite structures should be very important and can be further studied in future work.

## Figures and Tables

**Table 1:** The corresponding thermal analysis results of the PBT–EVOH polymer composite film using no triallylamine crosslinking agent.

Conversion (%)	Heating Rate (°C/min)			*Ea* (kJ/mol)	Slope	*R^2^*
	5	10	20			
	T (°C)					
5	339	359	376	211	11.58	0.999
10	345	378	396	151	8.30	0.965
20	350	382	407	142	7.80	0.991
30	353	384	415	134	7.37	0.999
40	356	386	420	131	7.22	0.999
50	358	388	425	127	6.97	0.999
60	361	391	430	124	6.83	0.998
70	364	394	435	122	6.69	0.996
80	372	398	441	125	6.87	0.997

*R^2^* is correlation coefficient.

**Table 2:** The corresponding thermal analysis results of the 0.2%-triallylamine-crosslinking PBT–EVOH polymer composite film.

Conversion (%)	Heating Rate (°C/min)			*Ea* (kJ/mol)	Slope	*R^2^*
	5	10	20			
	T (°C)					
5	354	370	391	209	11.47	0.996
10	368	383	403	224	12.29	0.995
20	380	395	415	227	12.49	0.995
30	387	403	422	230	12.65	0.999
40	393	408	428	232	12.72	0.995
50	399	413	434	232	12.76	0.990
60	405	419	441	228	12.51	0.990
70	411	426	447	231	12.68	0.993
80	420	433	455	237	13.03	0.982

*R^2^* is correlation coefficient.

**Table 3:** The corresponding thermal analysis results of the 1.5%-triallylamine-crosslinking PBT–EVOH polymer composite film.

Conversion (%)	Heating Rate (°C/min)			*Ea* (kJ/mol)	Slope	*R^2^*
	5	10	20			
	T (°C)					
5	350	363	379	259	14.21	0.998
10	372	384	398	296	16.26	0.999
20	385	397	410	312	17.15	0.999
30	392	404	418	304	16.69	0.999
40	398	409	424	305	16.76	0.994
50	403	415	430	297	16.34	0.997
60	409	420	435	310	17.01	0.994
70	416	427	442	313	17.17	0.994
80	425	435	449	340	18.69	0.993

*R^2^* is correlation coefficient.

**Table 4:** Ionic conductivity analysis results of the PBT–EVOH polymer composite electrolyte membranes.

Triallylamine Content (%)	Time (h)	*L* (cm)	*R_b_* (Ω)	Area (cm^2^)	*σ* (S cm^−1^)
0.0	24	0.0097	15.78	1.29	4.77 × 10^−4^
0.2	24	0.0106	4.67	1.29	1.76 × 10^−3^
1.5	24	0.0105	9.74	1.29	8.36 × 10^−4^
0.0	48	0.0100	1.88	1.29	4.12 × 10^−3^
0.2	48	0.0108	1.97	1.29	4.25 × 10^−3^
1.5	48	0.0095	1.46	1.29	5.04 × 10^−3^

## Data Availability

Not applicable.
